# Searching for the perfect goalkeeping personality. Myth or reality?

**DOI:** 10.3389/fpsyg.2024.1418004

**Published:** 2024-07-29

**Authors:** Jan Spielmann, Fabian Otte, Tom Schumacher, Jan Mayer, Stefanie Klatt

**Affiliations:** ^1^Department of Sports Sciences, Saarland University, Saarbrücken, Germany; ^2^TSG ResearchLab, Zuzenhausen, Germany; ^3^Borussia Mönchengladbach, Mönchengladbach, Germany; ^4^U.S. Soccer Federation (USSF), Chicago, IL, United States; ^5^Institute of Exercise Training and Sport Informatics, German Sport University, Cologne, Germany; ^6^TSG Hoffenheim, Zuzenhausen, Germany

**Keywords:** personality, big-five, football, soccer, athletes

## Abstract

**Background:**

Psychological factors such as personality characteristics are influential factors of the goalkeeping performance in football (soccer). Not only for individualized treatment in practice, also from a scientific point of view, profiling goalkeepers is a relevant part of understanding athletes. The aim of this study was to investigate personality traits of goalkeepers of different expertise, age, and sex.

**Methods:**

Using the Five Factor Model of personality we assessed personality traits of 132 male and female football goalkeepers ranging from youth to senior and low to elite level. A series of analysis investigated differences between the groups focusing on expertise, age, and sex.

**Results:**

Significant differences in the personality trait agreeableness between groups of different expertise and sex could be detected. Although a significant difference in neuroticism levels of males and females could be shown.

**Conclusion:**

This study was a first step of profiling football goalkeepers of different expertise, age, and sex. The study calls for more replication in this specific field of football and goalkeeping in general to understand the influence of personality characteristics on sport performance.

## Introduction

“*Goalkeepers need an element of insanity. Who else would want to stand there and allow people to shoot balls at their face or abdomen, and still think it's great?”*—Oliver Kahn, three-time winner of the IFFHS world's best goalkeeper award.

[cited from Gorris and Kubjuweit (2008)]

The narrative around football goalkeepers (GK) is often linked to a presentation of distinct psychological profiles with “strong” personalities that also may be perceived as “outside the norm” or, more jokingly, “not quite right” (Giertz, 2014). From a scientific point of view, current research does not provide conclusive answers to the question if top-level GKs generally differ in their personality profiles from those with lower performance levels. While, to date, there have only been few studies investigating GKs' personality traits, it remains largely unclear to what extent professional GKs embody certain personality characteristics. Empirical knowledge about potentially more dominant personality characteristics in professional GKs (compared with their rather less advanced counterparts), across male and female GKs at varying performance levels, could largely influence talent identification and scouting processes. Due to the lack of knowledge in this field and in order to support psychological consulting, training, and personality development, the following study investigates the existence of “*a perfect goalkeeping personality profile”* for performance at the professional level. We aim to examine whether this idea is close to reality or, rather, a full-on myth. For one, potential results indicating existence of an “idealized personality profile” for GKs at the professional level would assist researchers, psychologist and coaching practitioners in searching for certain personality traits when identifying and developing the future likes of world-class GKs, such as Mary Earps, Merle Frohms, Alisson Becker or Yann Sommer. In contrast, potential dispersed findings could make a case for an individual-environment-centered coaching approach (Otte et al., 2021). This coaching perspective equally considers and entangles (i) individuality of and differences between each GK, independent of performance level, experience and gender; and (ii) the development and performance contexts that players are embedded into (Sullivan et al., 2021).

Finally, prior to diving into the presented research study, the following paragraphs provide deeper theoretical understanding into positional demands in football goalkeeping and current empirical knowledge about personality profiling in sports and its connections with athletic expertise and gender differences.

### Research on positional demands in football goalkeeping

Concerning the positional requirements, goalkeeping in football arguably demands different skills that go beyond those of outfield players, not only from a tactical-technical point of view (for detailed overviews see Rechner and Memmert, 2010; Otte et al., 2022). In brief, the majority of the (limited number of) studies on goalkeeping deal with topics, such as physiological performance data on GKs' body composition, jumping power, sprint values (Sporis et al., 2009; Gil et al., 2014; Rebelo-Gonçalves et al., 2015), GKs' physical training loadings (e.g., White et al., 2020), position-specific behavior (Memmert et al., 2013; van der Kamp et al., 2018; Navia et al., 2019), GK-specific skill training periodization and coaching (Otte et al., 2019, 2020a,b), and perceptual-cognitive abilities (Savelsbergh et al., 2002; Woolley et al., 2015).

From a sport psychological standpoint, GKs are confronted with exceptional and distinctive challenges (West, 2018). For instance, a GK's game performance often is rated by an extreme dichotomy of either a successful or poor performance, which can be seen nearly every weekend: One save or, contrastingly, one goalkeeping mistake potentially determining the whole GKs rating. Thus, in professional football the outside perspective of fans, spectators, media, and other external parties seemingly has little room for gray areas. This leads to increased pressures for GKs to perform or, more drastically, to avoid mistakes. Put simple, the specific role of the GK in football appears highly demanding from a mental perspective and therefore, requires a stress-resistant psychological profile (Otte et al., 2020c). The classical psychological field of personality research appears to be relatively underrepresented, although relevance is obvious: utilizing a comprehensive approach, Hughes et al. (2012) emphasize the importance of the categories of concentration, motivation, attitude, and body language when evaluating GKs. These categories may coherently be combined with the results of a recent study on GK training and the requirement profile for professional GKs (Otte et al., 2019). In their qualitative study, the authors asked professional goalkeeping coaches to holistically reflect on the question of: “What critical skills does a top goalkeeper need?”. Among numerous physical and tactical-technical factors, the interviewed experts highlighted the area of “mentality” as an essential component in high-performance goalkeeping. Using keywords, such as “courage”, “concentration”, “work attitude and professionalism”, coaches stressed the relevance of mental skills and a distinct GK “personality”. Interestingly, it is precisely the latter term of “personality” that again bridges the gap to this research, analysis, and evaluation of personality traits of GKs. Finally, due to a lack of research on personality profiling in football (here, goalkeeping), this paper aims to investigate differences in personality traits of GKs on different performance levels (i.e., professional, semi-professional/amateur, and elite-youth GKs) and potential gender differences between male and female GKs. Current theoretical and scientific knowledge within the field of personality research in sport will be presented in the following paragraphs and later re-connected to the football goalkeeping context.

### Current scientific knowledge about personality profiling in sports

#### Personality and sports

Personality can be assessed by the use of trait assessments. Differential psychology often uses the Five Factor Model of Personality (FFM; McCrae and Costa, 1999; Mc Crae and Costa, 2008), which can be associated with a wide acceptance throughout literature (de Moor et al., 2012; Allen et al., 2013; Bircher et al., 2017). It divides personality into five traits: openness (O; curious, creative, and imaginative), conscientiousness (C; organized, punctual, and structured), extraversion (E; sociable, outgoing, and active), agreeableness (A; good-natured, unselfish, and forgiving), and neuroticism (N; anxious, hostile, and irritable). Besides scientific interest in assessments of personality traits, practical deductions can be used for everyday work. Therefore, scientific assessments can always provide objective perspectives of somebody's needs and motives as an addition to subjective estimations. Specifically in the world of high-performance sports, latter form of subjective estimations is overrepresented when it comes to talent identification, individualized action and developing processes (Cripps et al., 2019). Applied working personnel like sport psychologists and coaches can benefit from conclusions of an athlete's personality expression in terms of individualized intervention, consulting, coaching, and training. Depending on a certain characteristic or expression, communication and course of action should be adapted to each individual to provide best fittings possible. For example, literature shows beneficial interdependences between knowledge about athletes personality characteristics and important personal and career transitions (Laurin, 2009), integration processes (Beauchamps et al., 2007), and interpersonal relationships (Cuperman and Ickes, 2009; Jackson et al., 2011; Allen et al., 2013). Further, players can benefit from confronting themselves with their own trait-profile as an instrument of personality-development and setting-specific orientation. This could influence diverse factors of an athlete's life like training structuring (conscientiousness), risky decision making (neuroticism), diversify processes (openness), self-centration (agreeableness), or relationship building (extraversion), which at best leads to enhanced player long-term development and improved performances, both on and off the pitch (Piedmont et al., 1999; García-Naveira et al., 2011.; Ruiz-Barquín and García-Naveira, 2013).

Additionally, several hypothesis and theories have been developed over the years to better understand the relationship between sports and personality. To further analyze the findings of this study, we also give a broad overview to these theories. One crucial distinction hereby is the difference between the “development hypothesis” and the “selection hypothesis”. Proponents of the development hypothesis argue that sport activity influences the athlete's personality, while proponents of the selection hypothesis argue that the influence is the other way around—personality characteristics make athletes choose certain sports (García-Naveira and Ruiz-Barquín, 2016).

In general, both hypotheses can be combined in a mixed approach, as the selection and active participation in a sport both influence an individual's psychological profile sports (García-Naveira and Ruiz-Barquín, 2016). This lines up with the theory of “performance hypothesis”. The performance hypothesis, developed by García-Naveira and Ruiz-Barquín (2016), argue that certain personality traits are inherently linked to the heightened performance in a sporting context. As an example, could goalkeepers which personality type is considered extroverted, adapt more easily to the demands of the position compared to introverted ones and therefore play on a higher level? The performance hypothesis would agree to said question, which could theoretically allow a personality distinction between different levels of expertise in relevant sport positions.

#### Personality and athletic expertise

Personality characteristics of individuals and groups representing high expertise levels in any field of interest are often in focus of research; this, simply because these individuals have something unique, special and often the ability to do things “regular” humans are not capable of. For example, researchers investigated personality profiles of Mount Everest climbers (Egan and Stelmack, 2003), Olympic athletes (Piepiora et al., 2022b), or ultra-marathon participants (Hughes et al., 2003). As mentioned above, such an exposed role can also be applied to high-level football goalkeeping. Digging deeper into this specific clientele, it is worth using a bottom up approach by reviewing findings outside the goalkeeping field: focusing on the basic levels of physical activity, meta-analysis found positive correlations with extraversion, conscientiousness (Rhodes and Smith, 2006; Wilson and Dishman, 2015) and openness (Wilson and Dishman, 2015), whereas neuroticism was associated negatively (Rhodes and Smith, 2006; Wilson and Dishman, 2015). Studies focusing on the bidirectional associations between the constructs are also worth to be highlighted (Tolea et al., 2012; Stephan et al., 2014; Allen et al., 2015). For example, Allen et al. (2017) could show, that personality has a relevant impact for change in physical activity, whereas physical activity is relatively unimportant for changing personality characteristics. Classifying these general considerations into expertise levels, there are other contexts (e.g., occasional or academia settings), in which personality has been proven to influence on domain-specific success (Poropat, 2009; Furnham, 2018). Similar results can be reported for the setting of sports.

There is an increased number of studies focusing on the role of personality on athletic expertise and success. Examples for this field are investigations of differences in personality profiles of selected and non-selected athletes for the Paralympics (Martin et al., 2011), athletes' match statistics throughout a season (Piedmont et al., 1999), and personality characteristics as a prediction criteria for expertise (Morgan and Johnson, 1978; Aidman, 2007; Martin et al., 2011). When examining expertise levels in sports, high-level athletes show lower expressions for neuroticism (e.g., Kirkcaldy, 1982; Allen et al., 2011; Steca et al., 2018; Vaughan and Edwards, 2020), and higher expressions for extraversion (e.g., Williams and Parkin, 1980; Newcombe and Boyle, 1995; Egloff and Gruhn, 1996), conscientiousness (e.g., Allen et al., 2011; Steca et al., 2018; Vaughan and Edwards, 2020), and openness (e.g., Goddard et al., 2019; Vaughan and Edwards, 2020). Results for agreeableness remain unclear, as both higher (Allen et al., 2011) and lower (Vaughan and Edwards, 2020) expressions have been found. Another approach is operationalizing expertise by age progression, as older athletes (in comparison to younger athletes) proved their ability to perform on a specific level for a longer period of time. From a longitudinal point of view, the affiliation to a certain stage of expertise is less influenced by short term specific biases like performance peeks, over- or underrating, and luck. Those examined athletes demonstrated their ability against all possible odds throughout their career. Here, one study investigating young and senior athletes showed larger expressions for agreeableness, conscientiousness, and openness in the latter group (Trninić et al., 2016). This could support the approach of using age as a potential variable defining expertise, as at least conscientiousness and openness (as mentioned above) differentiate higher- from lower-level athletes. As specific characteristics and combinations of traits could be beneficial for different sports or expertise levels, these findings should always be interpreted considering their specific settings. As most of the current studies use samples of various disciplines representing various population sizes, profile requirements, and levels of professionalism, the mentioned findings are not generally transferable. To clarify, whether or not these trends of expertise levels are applicable to one specific discipline and playing position (i.e., football goalkeeping), this study aims to further investigate.

#### Personality and gender differences

The popularity of female sport is an obvious and increasing process of modern sport development, specifically in football. For example, the European Women's Championships (Women's EURO) made a progression in global audience from 116 million (2013) to 178 million (2017) to 365 million in the tournament of 2022 in England (UEFA, 2022). Although the popularity of female football is rising, women are still facing barriers such as lack of funding or basic concerns like finding suitable teams (O'Reilly et al., 2018). Similar circumstances can be found in the scientific world (Emmonds et al., 2019): female-specific research is dragging behind because of long-term inequality like distribution of resources which goes in line with levels of professionalism and participation. In this line, the field of goalkeeping is definitely not an exception.

Personality differences between males and females are one big field of interest for differential psychology. For norm populations, males tend to have lower levels of conscientiousness, neuroticism, agreeableness, and extraversion (Feingold, 1994; Costa et al., 2001; Schmitt et al., 2008). There is some evidence, that these findings could be transferred to the sporting context. For example, some researchers are of the opinion that physically active females display personality characteristics closer to males than inactive females (Fleming, 1934; Williams and Parkin, 1980; Allen et al., 2013). Nevertheless, Allen et al. (2011) found males scoring lower in conscientiousness, neuroticism, and agreeableness in a sample of different expertise levels and sports. Later, Gyomber et al. (2013) showed lower scores for extraversion and openness in male than in female subjects. It is suggested, that those findings could be directly transferred to expressions found in comparisons between male athletes and non-athletic populations (Allen et al., 2013). Notably, compared to research outside sports, these findings are no more than trends, as there are also contrary results published (O'Sullivan et al., 1998; Rhodes and Smith, 2006; Sutin et al., 2016). The only trait which seems in line throughout most findings is neuroticism showing higher expressions for females in general (Kirkcaldy, 1982; Colley et al., 1985; Newcombe and Boyle, 1995; Ruiz-Barquín, 2005). Like in other scientific areas, further research to investigate general gender differences in athletic populations, specifically in high-level athlete samples, is needed.

### Aims and hypotheses

This study aims to investigate personality traits of a sample of football GKs with the Five-Factor Model. In detail, differences in trait-characteristics of various expertise and age groups together with a gender separation are point of interest. It is hypothesized that GKs of higher expertise levels show larger expressions of extraversion, conscientiousness, and openness and lower values in neuroticism than GKs of lower expertise levels (hypothesis 1). Regarding gender, it is assumed that female GKs show higher values for neuroticism than male GKs (hypothesis 2). Furthermore, we hypothesize that as female GKs progress in expertise, their neuroticism values will be closer to the lower expertise male GKs (hypothesis 3).

## Methods and materials

### Participants

In total, 132 football goalkeepers (96 male; 36 female) aged 16–37 years (*M* = 20.43 years, *SD* = 4.94) participated in this study (Table 1). All participants were German native speakers to prevent the dataset of biases such as misunderstanding the questionnaires or test instructions. In sum, all GKs were current players of 38 different clubs all over Germany, ranging from the U17's to senior level. Altogether, 37 GKs (28.03%) have been or were part of a youth or adult national team. Regarding our hypothesis, we ran several *post-hoc* analyses with the program G^*^Power (Version 3; Faul et al., 2007) to retrospectively determine the Power of our dataset. For hypothesis 1, we achieved a Power of 0.942 with a Pillai's V of 0.15. Hypothesis 2 had a Power of 0.999 with a Pillai's V of 0.255 and hypothesis 3 had a Power of 0.999 with a Pillai's V of 0.485.

**Table 1:** Descriptive NEO-FFI statistics (*n* = 132, plus gender and level separation, raw scores).

	**Level**		**Trait**				
			**N**	**E**	**O**	**A**	**C**
All athletes (*n* = 132)	Pro	M	15.79	30.28	26.34	34.86	37.28
	SD	5.75	5.09	4.79	3.99	5.81
	Elite youth	M	15.17	30.91	24.97	31.37	38.26
SD	6.46	4.49	5.34	4.85	4.48
Semi-pro/amateur	M	16.71	30.67	26.29	32.19	35.81
SD	7.48	3.44	5.28	4.92	5.67
Males (*n* = 96)	Pro	M	13.62	29.31	25.15	33.77	40.08
	SD	4.50	6.50	4.26	4.78	4.89
	Elite youth	M	14.56	30.67	24.98	30.33	37.72
SD	6.70	4.61	5.26	4.93	4.70
Semi-pro/amateur	M	16.11	30.22	26.33	31.33	35.22
SD	6.50	3.49	5.69	4.54	5.68
Amateur youth	M	15.00	30.25	24.42	31.25	36.00
SD	4.65	6.41	5.09	4.80	6.07
Females (*n* = 36)	Pro	M	17.56	31.06	27.31	35.75	35.00
	SD	6.16	3.62	5.11	3.09	5.62
	Elite youth	M	17.25	31.75	24.94	34.88	40.06
SD	5.24	4.09	5.80	2.28	3.13
Semi-pro/amateur	M	20.33	33.33	26.00	37.33	39.33
SD	13.31	1.53	2.00	4.51	5.03

### Personality assessment

The German adaptation by Borkenau and Ostendorf (2008) of McCrae and Costa's (1987) NEO-FFI questionnaire was used to determine athletes' personality traits. The questionnaire consists of 60 items rated on a five-point Likert scale (strongly disagree, disagree, neutral, agree, strongly agree). It is a self-report measure that assesses the five personality dimensions: extraversion (E), neuroticism (N), openness (O), agreeableness (A), and conscientiousness (C). The NEO-FFI is a well-established questionnaire with quality criteria reported in various populations (McCrae and Costa, 2004), especially in elite football players (Spielmann et al., 2022). Furthermore, reliability coefficients for the NEO-FFI in the current sample were N = 0.81, E = 0.66, O = 0.67, A = 0.69, and C = 0.81.

### Procedure

Prior to the commencement of this study, informed consent from all athletes (and a legal guardian for all participants under 18 years of age) was received, and the Institutional Ethics Committee approved this study (approval number: 19-19). Players answered the personality questionnaire via an online survey. The assessment had a standardized introduction and familiarization protocol, and a sport psychologist could always be consulted. Before the participants started, they were informed, that all results would stay anonymous, and they will not get any negative consequence if they do not participate. They did not get any compensation for being part of the study. The online survey was either presented during the professional clubs' standardized sport psychological diagnostics battery or sent directly in terms of personal contact. In the former case, the survey was answered in small group settings in a separate room. In the latter case, the survey was answered in an individual environment. Reading and answering the assessment took ~15 min. Finally, GKs' statements about their current and past playing levels were used to create participant groupings for statistical analysis. Using an applied approach based on football knowledge about the German senior and youth league systems and playing levels, six groups and their respective selection criteria were established (Supplementary Table 1).

### Statistical analysis

For most of the hypotheses a MANOVA with a protected F-Approach was used. The effects were subsequently controlled with the usage of a *post-hoc* Tukey Test. To analyze possible differences for effects of gender, *post-hoc* tests were conducted using a student's *t*-test. For the last hypothesis, we also used multiple pairwise comparisons to obtain specific differences between our diverse goalkeeper groups. The significance level was set at *p* < 0.05, and an estimate precision was provided using Wald- based 95% confidence intervals. Prior to the analysis, the data were first screened for outliers, missing data, and checked for normality using visual inspection of box plots through a Shapiro-Wilk test of normality in accordance to Tabachnick and Fidell (2014). Bonferroni correction was used to adjust α with a new level of α = 0.01.

## Results

### Preliminary analysis

All studies were preliminary checked for their assumptions. Due to the highly specialized sample size of elite athletes, certain outliers were noticeable and problems regarding univariate, especially in regarding the personality trait of Neuroticism. This unusually large distribution of values may be of interest when considering future analysis but may be due to the unique sample size. A removal of the factor Neuroticism resulted in no changes regarding the significance of the analysis and therefore remained in the analysis. Due to some of the preliminary assumptions being violated, the authors opted out to use Pillai's trace in the MANOVA analysis. This is because of the high robustness regarding violations of assumptions (Pillai, 1955).

### Expertise related differences

**Figure 1:**
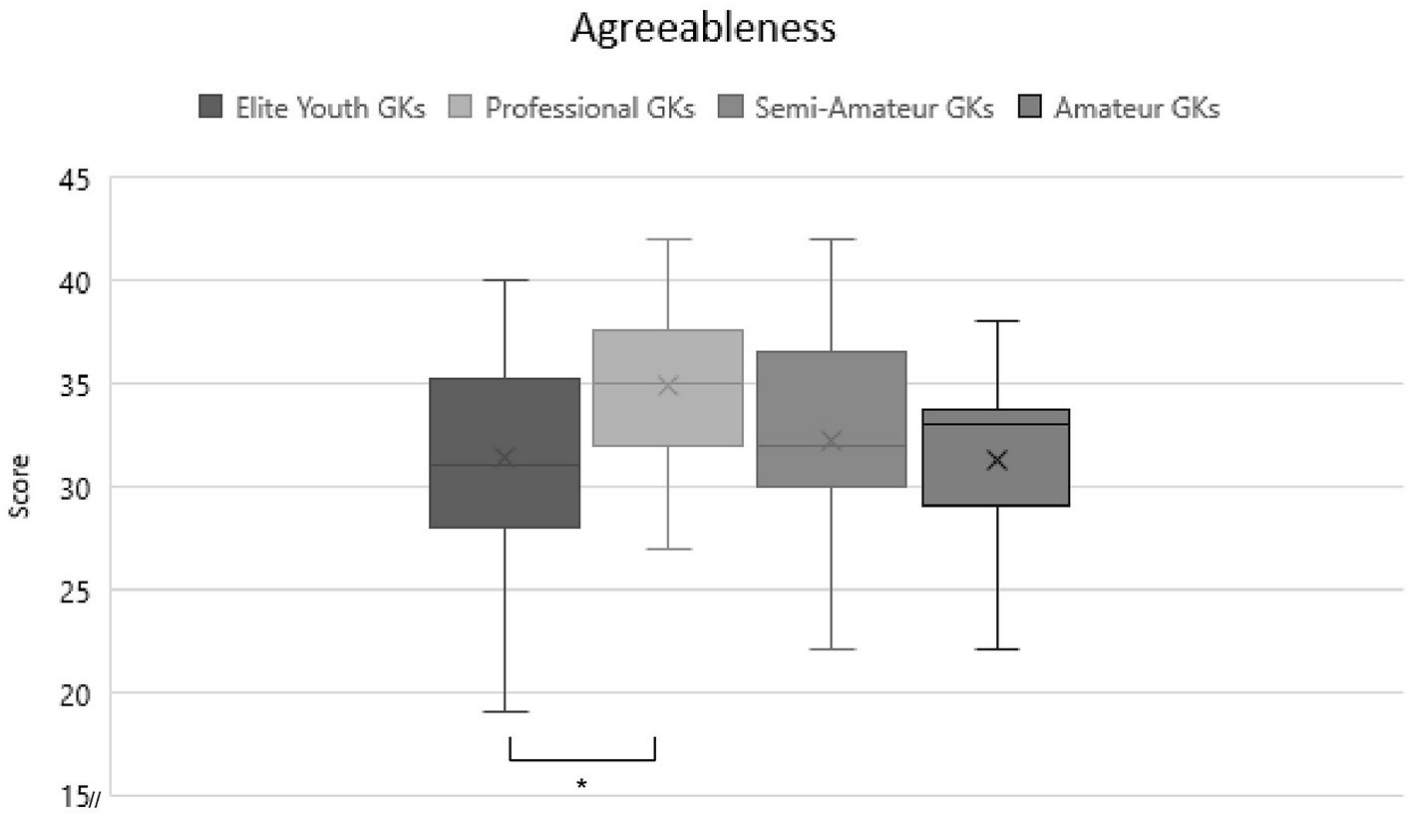
Comparison of agreeableness scores between expertise levels. Significant differences are marked with a*.

The first objective of the study was to examine differences in personality characteristics depending on expertise level and age, respectively. The MANOVA was significant at *F*_(5)_ = 4.045, *p* = 0.002, η^2^ = 0.138. As the five personality values were compared with each other, a Bonferroni correction in the singular ANOVA with a new critical α of 0.01 was used. This value was only reached by agreeableness with *F*_(3)_ = 3.983, *p* = 0.009, η^2^ = 0.085. This effect size indicates a medium effect. *Post-hoc* analysis using Bonferroni were done to clarify these results. As shown in Figure 1, they showed a significant difference between elite youth (*M* = 31.43, *SD* = 4.91) and pro GKs (*M* = 34.86, *SD* = 4.06). This indicates that pro GKs have a higher agreeableness score than elite youth GKs. No significant differences were found for the other personality traits or for the amateur groups and thus, hypothesis 1 is rejected.

### Gender related differences

**Figure 2:**
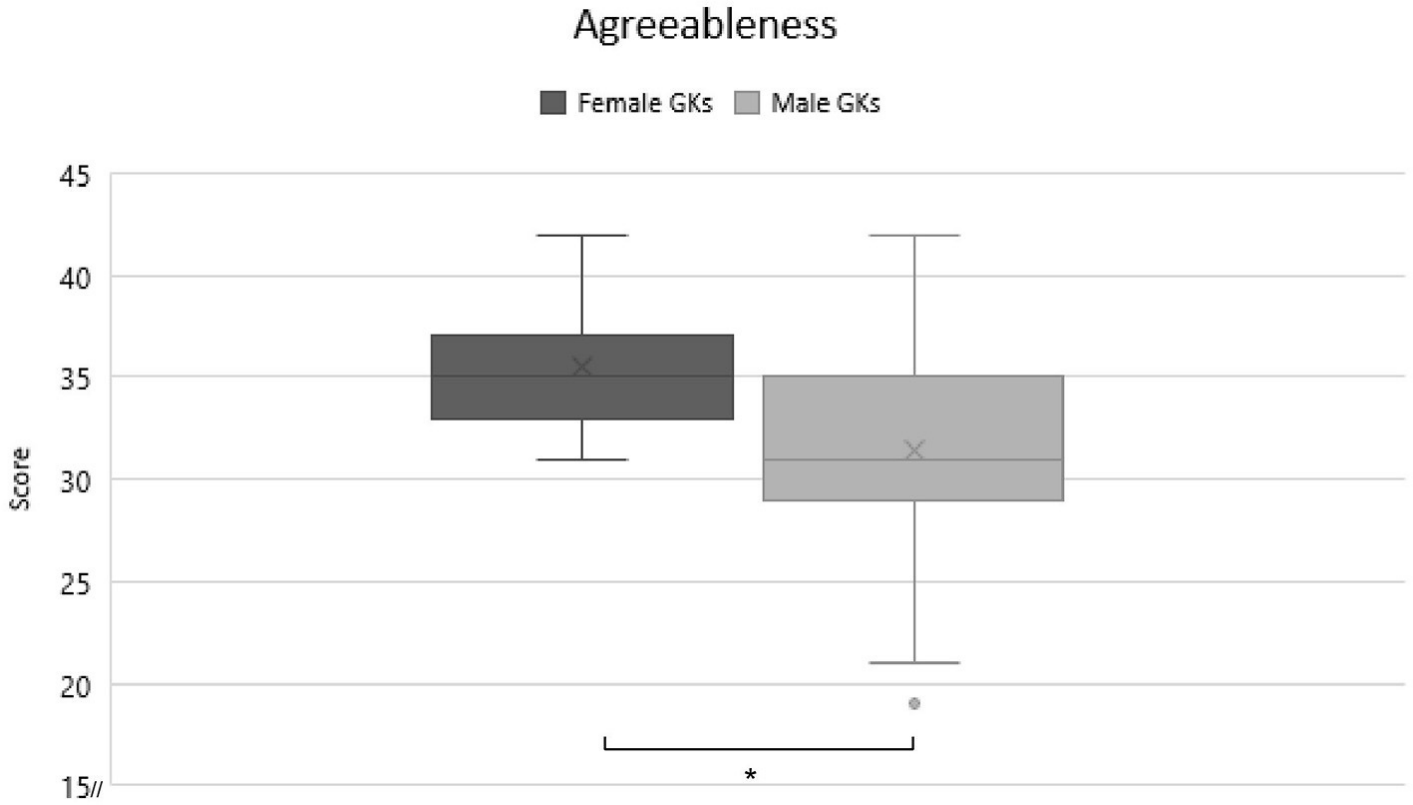
Comparison of agreeableness scores between female and male GKs. Significant differences are marked with a*.

**Figure 3:**
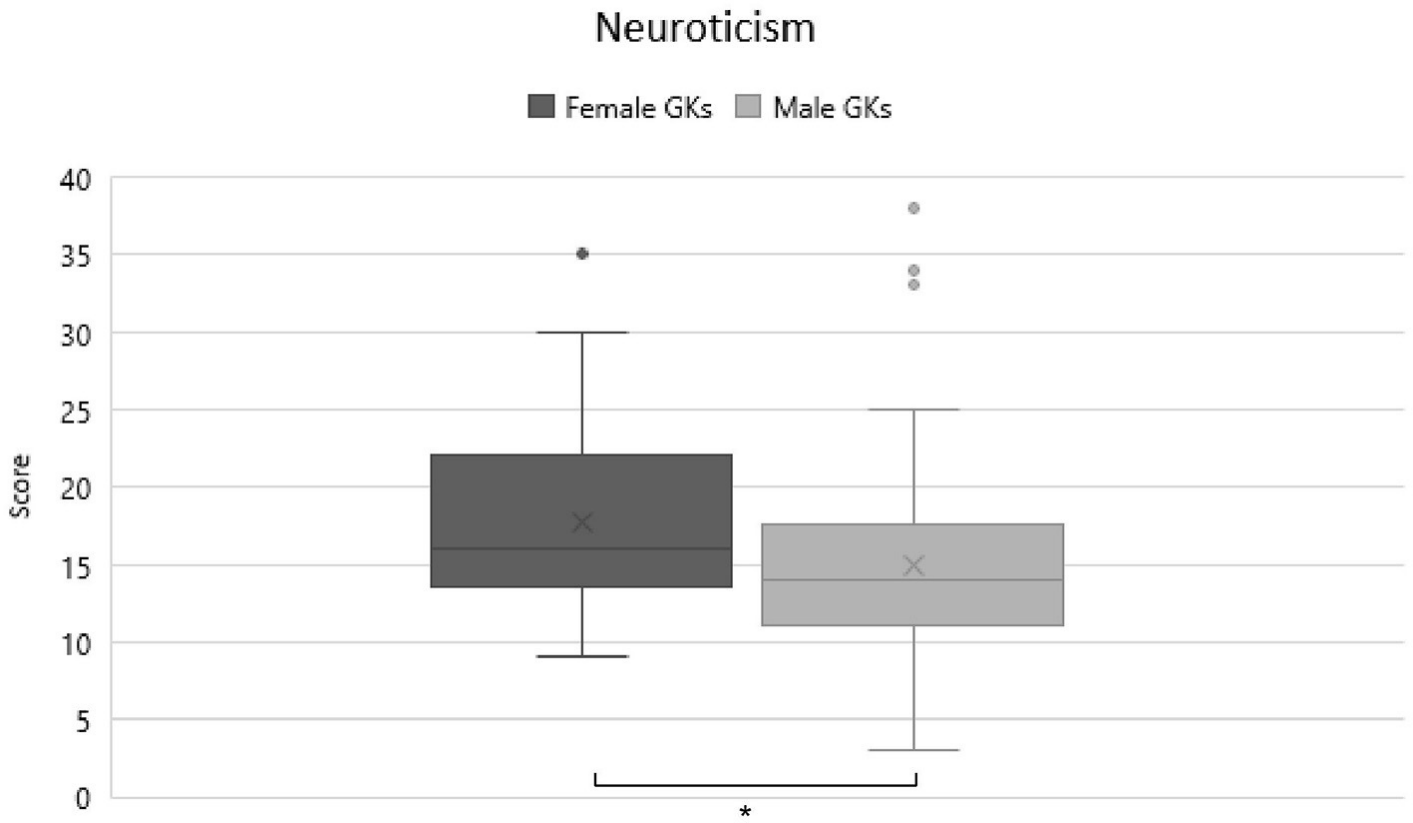
Comparison of neuroticism scores between female and male GKs. Significant differences are marked with a*.

As a second objective, differences between genders were determined. A MANOVA revealed a significant finding at *F*_(5)_ = 8.372, *p* < 0.001, η^2^ = 0.249. Additional ANOVAS according to protected F-Measure were performed to find exact difference. These showed, after Bonferroni correction, a trend in gender differences for neuroticism [*F*_(1)_ = 5.550, *p* = 0.02, η^2^ = 0.041] and significant gender differences for agreeableness [*F*_(1)_ = 24.865, *p* < 0.001, η^2^ = 0.161] scores (α = 0.01). Further *t*-tests were used to clarify the differences. Significant findings could be shown for neuroticism [*t*_(130)_ =2.328, *p* = 0.023, *d* = 0.04] and agreeableness [*t*_(130)_ =4.987, *p* < 0.001, *d* = 0.088]. In detail male GKs scored lower in both agreeableness (*M* = 31.09; *SD* = 4.912 vs. *M* = 35.49; *SD* = 2.86; Figure 2), and neuroticism (*M* = 14–77; *SD* = 6.17 vs. *M* = 17.66; *SD* = 6.32; Figure 3) as female GKs. The low effect sizes in this analysis could stem from the fact that we analyzed two samples with very different sizes. To obtain a higher effect size, future studies with more female goalkeepers should be conducted to fully understand possible personality differences between male and female GKs.

### Expertise and gender related differences

Lastly, the third objective of the study was to investigate if female GKs as progressing in expertise, their personality characteristics show closer comparability to lower expertise male GKs. The MANOVA itself was significant at *F*_(30)_ = 2.229, *p* < 0.001, η^2^ = 0.097. The additional ANOVAS showed significant effects for agreeableness [*F*_(6)_ = 5.082, *p* < 0.001, η^2^ = 0.205] and one less strong effect for conscientiousness [*F*_(6)_ = 2.323, *p* = 0.037, η^2^ = 0.106]. All of these effect sizes are generally deemed as medium—large. As hypothesis 3 focused on significant effects in neuroticism only, the assumption is rejected. After applying Bonferroni correction, conscientiousness is no longer significant, however this can be seen by the reason of the limiting sample size. Regardless and due to the intriguing sample, we will continue the analysis, however we must interpret conscientiousness findings with care.

**Figure 4:**
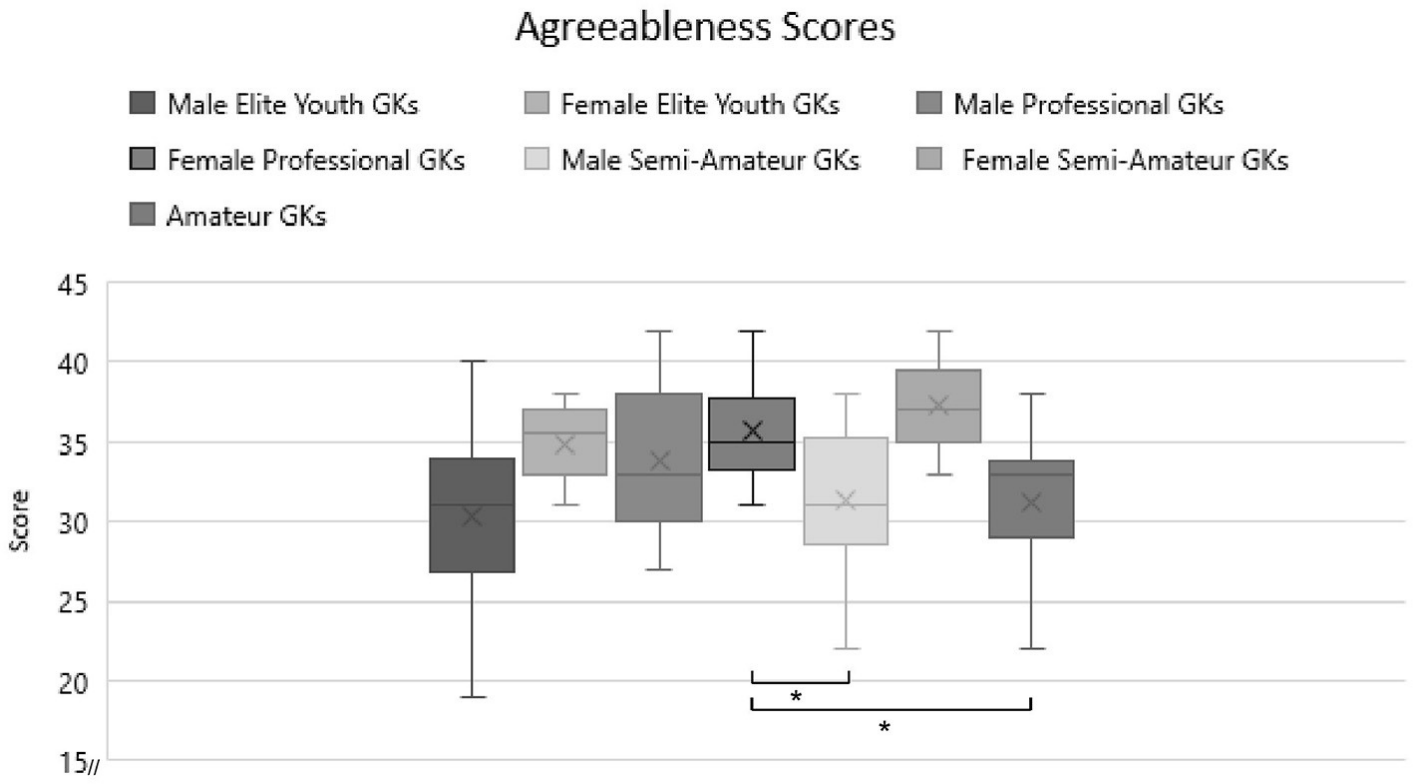
Comparison of agreeableness scores with expertise and gender separation. Significant differences are marked with a*.

Pairwise comparisons further analyzed the differences in agreeableness and conscientiousness. In agreeableness, male elite youth GKs have significantly lower agreeableness scores than female elite youth GKs (difference = −4.60, *p* < 0.001), male pro GKs (difference = −3.26, *p* = 0.021), female pro GKs (difference = −5.35, *p* < 0.001) and female semi-pro/amateur GKs (difference = −6.93, *p* = 0.009). Furthermore, female pro GKs have higher scores than male semi-pro/amateur GKs (difference = 3.265, *p* = 0.021) and male youth semi-pro/amateur GKs (difference = 3.750, *p* = 0.03). For an overview of these results, refer to Figure 4. The last finding is in line with hypothesis 3 in the way that the female group of highest expertise (pro GKs) show higher agreeableness scores than male groups of lower expertise (semi-pro/amateur).

**Figure 5:**
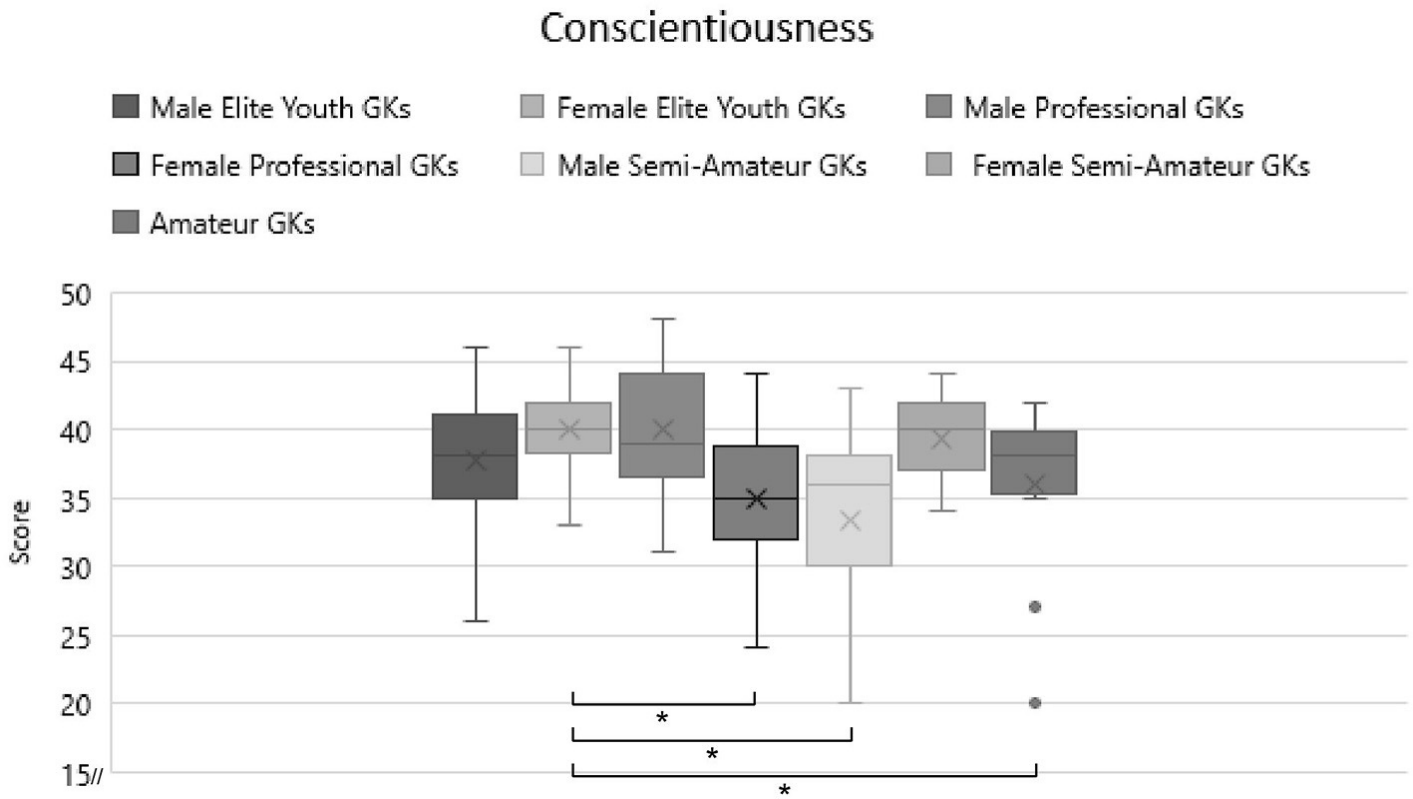
Comparison of conscientiousness scores with expertise and gender separation. Significant differences are marked with a*.

In conscientiousness, we can see that female elite youth goalkeepers have significantly higher values than female pro GKs (difference = 5.27, *p* = 0.003), as well as male semi-pro/amateur (difference = 3.77, *p* = 0.034) and youth semi-pro/amateur male GKs (difference = 4.27, *p* = 0.029). Additionally male pro GKs showed significantly higher scores in conscientiousness compared to female pro GKs (difference = 4.83, *p* = 0.011). For an overview of these results, refer to Figure 5.

## Discussion

Using the FFM of personality, the aim of the current study was to investigate personality traits of youth and adult football goalkeepers of various expertise levels, ranging from amateur to professional level. Furthermore, it was of interest to gain a more detailed view on gender-specific differences. Findings revealed heterogenous results for expertise levels and gender, mainly for the personality trait agreeableness, and are discussed from an individual-environment-centered perspective.

### Expertise related differences between senior professional and elite youth GKs

Interestingly, analysis only revealed a significant difference for the personality trait agreeableness. For differences in expertise, we initially suggested the exact opposite (hypothesis 1). From an individual-environment-centered focus on player development, the non-significant findings for hypothesis 1 (i.e., the prediction that higher expertise levels show larger extraversion, conscientiousness, and openness and lower values in neuroticism) appear remarkable. Since none of these personality traits seem to differentiate the group of professional goalkeepers from their younger or semi-pro/amateur counterparts, it could be interpreted as contrary to the performance hypothesis (García-Naveira and Ruiz-Barquín, 2016). Two main discussion points arise.

One, the often-cited notion of “context is everything” (Davids et al., 2021) for practice design and coaching transfers nicely to the domain of psychological GK profiling. Being aware of each player's individual context, constraints and socio-cultural background appears critical in performance sport. Clearly, every player must be regarded as a unique individuum that displays specific characteristics and demands; these, coaches and psychological support staff must recognize to individualize psychological development and maximize performance preparation. For example, when tasked to speak to a group of media representatives (i.e., a very common task for professional football players these days), a GK scoring high in openness and extraversion and low in neuroticism may feel and behave much differently about this scenario than a GK scoring lower/higher in these areas, respectively. In other words, only by understanding a GK's personality profile, coaches and psychologists may be able to support this (professional) player and assist in preparing for common events, such as media interviews, press conferences or likewise, in an individualized way.

Two, due to non-significant differences when comparing experts' personality traits with lower level or skillful GKs, it may be stated (by some) that using psychological diagnostics and profiling of individual GKs could be seen as an inefficient use of time and resources. However, we would argue the opposite: by profiling GKs' personality traits, practitioners within high-performance player development programs will be assisted in becoming aware of and understanding each individual GK's demands. In a recent investigation of coaches' views on their responsibilities regarding the coaching process and practice design, Selimi et al. (2023) emphasized the importance of relationship building with players and the coaches' initial responsibility of “developing people”. This idea aligns closely with our findings in a way that it appears invaluable for coaching and support staff within teams, clubs and national federations to gain in-depth understanding of each individual player's history and her personality traits. Here, use of standardized FFM of personality tests can be of instrumental help for practitioners.

Lastly, for our significant findings on agreeableness, in comparison to the other FFM traits, the status quo of current research is rather unclear. Nevertheless, our finding is in line with Allen et al. (2011) who also showed higher scores for agreeableness in higher level athletes. Thereby, we are in opposition to Vaughan and Edwards (2020). Using an approach where expertise is defined by age progression, a linkage to the studies of Trninić et al. (2016) and Piepiora et al. (2022a) is apparent and revealed similar results. From our view, different explanations could potentially underline this finding. Senior professional GKs, due to their numerous years of top-level playing and their “secure and stable” status within a club/team, may feel less under pressure to outperform competitors compared to youth elite GKs. In contrast, in an academy setting young GKs pursue the goal of signing a professional contract and hence, compete with an enlarged number of further GKs to achieve this aim; this, over time, could possibly lead to youth elite GKs displaying less agreeable behavior than their professional counterparts. Additionally, changing socio-cultural expectations, values and norms within modern-day societies have been shown to highlight stronger value-directedness toward elitism and individual competition (e.g., shown in younger generations in Swedish football; (Vaughan et al., 2022). Possibly regarding the trait of agreeableness, as much as this evolving value-directedness may shape skill development in football practice, it may also influence personality development and social behavior of aspiring elite footballers. Put simply, given the evolving socio-cultural constraints that influence and shape young adults when growing up, changes in personality traits toward less agreeable behaviors may be a consequence. Notably, this interpretation is strongly speculative and warrants further research.

### Gender related differences between male and female GKs

The primary results indicated that male GKs scored noticeably lower in agreeableness compared to their female counterparts. The disparities in neuroticism can only be considered a tendency due to the application of the Bonferroni correction. Despite this, it remains valuable to closely examine this particular insight. Our findings correspond to results from norm populations (Feingold, 1994; Costa et al., 2001; Schmitt et al., 2008). In sports, significant differences were shown for neuroticism (Kirkcaldy, 1982; Colley et al., 1985; Newcombe and Boyle, 1995; Allen et al., 2011) and agreeableness (Allen et al., 2011). The tendency for neuroticism could be explained by several reasons. First, the pure number of active athletes could lead to an increased selection effect in favor of football players with lower neuroticism, as it is associated with negative effects on athletic success (McKelvie et al., 2003; Piepiora, 2021) and mental health (De Moor et al., 2006). For example, the German Football Association (DFB) reports a number of 2.022.123 active male vs. 186.646 active female football players for the 2021/2022 season (DFB, 2023). Also, the still existing inequality of professionalism in terms of resources invested into coaching staffs and consulting (e.g., sport psychologists, psychotherapists, licensed coaches, etc.) could have an impact on neurotic behavior, like increased levels of anxiety or nervousness. Additionally, on a basis of masculine stereotypes (Chalabaev et al., 2013), neuroticism and its associations are yet interpreted as a sort of weakness (Sebbens et al., 2016). Leastwise, this bias appears with a higher quote in male football than in female settings.

The differences between male and female GKs in agreeableness are harder to explain as they are inconsistent in the sporting context. People with high levels of agreeableness tend to have higher standards in morality, sympathy, and cooperation. Like with neuroticism, the pressure in male football could favor athletes with lower levels of agreeable behavior. Also, as stated above, the professional system in football sometimes educates and forces youth athletes to show such a behavior when they need to always be the best, outperform others and be less compassionate (Beavan et al., 2022). This trend could even be stronger when it comes to special characters like GKs, where in most cases there is only one clear number one that needs to protect their status and position from potential rival candidates.

### Expertise and gender related differences

The subdivision of male and female groups showed male elite youth GKs scoring significantly lower in agreeableness. The finding could be a result of the aforementioned high pressures in this male age group, given that elite youth players play their final years in football academies with the hope of being awarded a senior professional contract, and the fear of having to transfer to semi-pro/amateur leagues or even end their ambitious careers. To showcase oneself in the best way possible, an aspiring youth elite GK may be well-aware of the situation that all manageable aspects in their last years of academy football may influence chances of becoming a professional or not. This awareness could result in a behavior which is informed by egocentricity and suspiciousness, even if that can be interpreted negative from an ethical standpoint. One explanation, why this finding could not be detected in the female elite youth group could be that female players pass through this transition period at a younger age. This has various reasons related to the organizational structure of female pro sport (specifically in Germany), being maybe the most influential aspect. For example, the second highest league in German senior female football (i.e., 2. Frauen-Bundesliga) consists approximately one half of first division clubs' reserve/U-21s teams. These “farm teams” mainly focus on highly talented young players, which are often allowed to still play in U-17s youth leagues. As strength density in those leagues is rather weak, clubs potentially elevate young female players earlier into senior teams than they would do with male football players. As the current study implemented GKs with an age of 16 plus, future research should also implement younger age groups of the highest performance level to dig deeper into male/female differences.

### Limitations and future directions

The current study should be considered in the context of some limitations that we would like to address. We decided to investigate personality traits of both male and female goalkeepers of various ages and expertise levels. As the circumstances under which male and female GKs are identified and developed can differ from rather equal to extreme, it is hard to compare these individual GKs and groups on specific characteristics. As literature-based grouping strategies could not be transferred to the field of goalkeeping, we tried to group the participants using an applied approach (Supplementary Table 1). This grouping strategy could arguably lead to different results dependent on whether a specific GK would be classified as a “professional”, “semi-professional” or “elite youth”. For example, some male football players can finance their lives with an affiliation to a club in the 5th division (i.e., amateur-level football according to our grouping), whereas female players often have a second mainstay besides playing first division football (i.e., still grouped as senior professional due to playing at the highest level).

Next, the overall sample size displays a limitation of our study, which can be seen in the interpretation of the personality trait conscientiousness after Bonferroni correction (hypothesis 3). Nevertheless, as we targeted the specific football position of the GK with significantly lesser player numbers compared to outfield players and managed to recruit an enlarged number of GKs playing at the highest performance level possible (e.g., the 1st German Bundesliga), we are convinced of the high-quality insights into an often called “closed door world” of professional football.

Moreover, it is important to mention, that only European German native speakers were assessed to prevent the dataset from misunderstanding biases. As the European academy system can differ from countries outside Europe, the findings should be transferred carefully.

### Practical applications

Assessing personality profiles in athletes has several practical applications for different peer groups. Our findings could show that there is no clear pattern that elevates an ambitious goalkeeper to a professional level. Incorporating age and gender diversity further complicates this generalized approach. Nevertheless, assessing and focusing on an athlete's personality characteristics is practically necessary to find the most suitable settings and provide a basis for sport psychological consulting. When athletes concentrate on their individual profiles, they are able to understand the interdependencies between relationships in both their personal (e.g., parents, partners, etc.) and their sporting contexts (e.g., coaches, teammates). By identifying similarities and differences, they can discover potential pathways for healthy and constructive circumstances, which could be a beneficial aspect of an athlete's life. Clubs, associations, and organizations can benefit from personality assessments for scouting purposes and to build suitable team cohesion. It is important to emphasize that such questionnaire-based instruments should not be used to identify “black sheep” in an existing team, but rather to identify missing characteristics that need to be recruited. The former approach would only lead to higher rates of social desirability and therefore miss the mark.

In the end, the strongest impact of personality profile assessments in practice is achieved when they are used as supportive instruments for all kinds of sport psychological work and not as (de)selection criteria. Their greatest benefit lies in using them to understand an athlete's characteristics in more detail and to help them find or build the most suitable setting for their individual potential development.

## Conclusion

In the current study, we aimed to investigate differences in personality traits of football goalkeepers. Compared to previous research, we used the well-established FFM to assess both male and female GKs of different age and expertise levels. Besides gender-specific differences, our findings were not in line with results of comparable studies focusing on expertise in the sporting context. From an individual-environment-centered coaching perspective, however, non-significant differences between various player groups and for some personality traits display invaluable findings. It appears critical for coaches to understand each individual player's context, constraints, and background. Hence, psychological profiling and consulting work remain highly beneficial to support individualized player development and coaching, at academy and senior levels, as well as in men's and women's football. In other words, results of this study would argue against the existence of “*an idealized goalkeeping personality profile”* for performance at the professional level. Thus, there appears to be no silver bullet and researchers, psychologists and coaching practitioners remain (positively) challenged when identifying and developing top-level GKs. Notably, as this research displays a first attempt at assessing personality traits of GKs, the interpretation and placement into the current scientific discourse has to be handled with caution. More research is encouraged and needed on whether (our first step into) studying personality traits of GKs is replicable, and to support both scientists and practitioners to generalize the current study's findings.

## Data availability statement

The raw data supporting the conclusions of this article will be made available by the authors, without undue reservation.

## Ethics statement

The studies involving humans were approved by Universität des Saarlandes Ethikkommission der Fakultät HW Campus A1 3 66123 Saarbrücken. The studies were conducted in accordance with the local legislation and institutional requirements. Written informed consent for participation in this study was provided by the participants' legal guardians/next of kin.

## Author contributions

JS: Conceptualization, Data curation, Formal analysis, Methodology, Project administration, Writing – original draft, Writing – review & editing. FO: Conceptualization, Writing – original draft. TS: Visualization, Writing – review & editing. JM: Supervision, Writing – review & editing. SK: Data curation, Formal analysis, Supervision, Writing – review & editing.
