# Supplementary material for: Searching for the perfect goalkeeping personality. Myth or reality?
Source: Front Psychol. 2024 Jul 29;15:1418004. doi: 10.3389/fpsyg.2024.1418004 (PMC11318172; doi:10.3389/fpsyg.2024.1418004)
Supplement: Supplementary file 1 [file Table_1.docx]

**Supplementary table 1**

Applied participant groupings based on expertise and playing levels within the German football structure.

| **Category** | **Criteria** | **Sample size (n)** |
| --- | --- | --- |
| Pro GK  (male) | Senior-pro GKs playing in either the German 1^st^, 2^nd^ or 3^rd^ division Men’s Bundesliga; including GKs over 23 years of age playing for reserve teams of German Bundesliga clubs. ~8 training sessions / week. | 13 |
| Pro GK  (female) | Senior-pro GKs playing in the German 1^st^ division Frauen-Bundesliga. ~7 training sessions / week. | 16 |
| Elite youth GK  (male) | GKs playing in farm/reserve (2^nd)^ teams of German first division clubs; in the German U17s/ U19s youth Bundesliga, or minimum, in the second highest youth division in an official certified academy. ~6 training sessions / week. | 54 |
| Elite youth GK (female) | GKs playing in either the German U17s youth Bundesliga or in the 2^nd^ Frauen-Bundesliga. ~5 training sessions / week. | 16 |
| Semi-pro/amateur GK (male) | Senior GKs playing below the German first three professional divisions (i.e., Regionalliga). ~ < 5 training sessions / week. | 18 |
| Semi-pro/amateur GK (female) | Senior GKs playing below the German 1^st^ division Frauen-Bundesliga; exception full-time senior first teams of clubs playing in the 2^nd^ Frauen-Bundesliga (i.e., then considered as senior pro). ~ < 5 training sessions / week. | 3 |
| Amateur youth GK  (male) | Youth GKs playing below the German 2^nd^ youth divisions + nonofficial certified academy players  (i.e., Regionalliga). ~ < 5 training sessions / week. | 12 |
| Total |  | 132 |
